# Genome-Wide Mutation Scoring for Machine-Learning-Based Antimicrobial Resistance Prediction

**DOI:** 10.3390/ijms222313049

**Published:** 2021-12-02

**Authors:** Peter Májek, Lukas Lüftinger, Stephan Beisken, Thomas Rattei, Arne Materna

**Affiliations:** 1 Ares Genetics GmbH, Vienna 1030, Austria; peter.majek@ares-genetics.com (P.M.); lukas.lueftinger@ares-genetics.com (L.L.); stephan.beisken@ares-genetics.com (S.B.); 2 Department of Computational Systems Biology, University of Vienna, Vienna 1030, Austria; 3 Centre for Microbiology and Environmental Systems Science, Division of Computational Systems Biology, University of Vienna, Vienna 1030, Austria; thomas.rattei@univie.ac.at

**Keywords:** machine learning, genomics, antimicrobial resistance, antibiotics, WGS, genome-wide mutation scoring

## Abstract

The prediction of antimicrobial resistance (AMR) based on genomic information can improve patient outcomes. Genetic mechanisms have been shown to explain AMR with accuracies in line with standard microbiology laboratory testing. To translate genetic mechanisms into phenotypic AMR, machine learning has been successfully applied. AMR machine learning models typically use nucleotide k-mer counts to represent genomic sequences. While k-mer representation efficiently captures sequence variation, it also results in high-dimensional and sparse data. With limited training data available, achieving acceptable model performance or model interpretability is challenging. In this study, we explore the utility of feature engineering with several biologically relevant signals. We propose to predict the functional impact of observed mutations with PROVEAN to use the predicted impact as a new feature for each protein in an organism’s proteome. The addition of the new features was tested on a total of 19,521 isolates across nine clinically relevant pathogens and 30 different antibiotics. The new features significantly improved the predictive performance of trained AMR models for *Pseudomonas aeruginosa*, *Citrobacter freundii*, and *Escherichia coli*. The balanced accuracy of the respective models of those three pathogens improved by 6.0% on average.

## 1. Introduction

Antibiotics are small molecules with bacteriostatic or bactericidal activity that are used to treat bacterial infections. The overuse of antibiotics has over time accelerated the emergence and spread of antimicrobial resistance (AMR) in bacteria [1]. Accurate antimicrobial susceptibility testing (AST) results are crucial to guide patient treatment as well as to inform antibiotic stewardship and outbreak monitoring. In this context, resistance predictions from whole pathogen genomes have the potential to complement or even replace phenotypic AST [2,3,4,5]. In recent years, various in silico methods have been applied to predict resistance phenotypes from whole genome sequencing data (WGS-AST) [6,7].

In principle, approaches for WGS-AST are either rules-based or machine learning (ML) based. The rules-based approach relies on the collection and curation of expert knowledge about AMR markers, e.g., genes and sequence mutations [8,9,10,11,12,13]. AMR prediction on a sequenced isolate is then performed by searching the isolate’s genomic sequence for the presence of AMR markers and interpreting the findings. This approach offers high clinical interpretability [6], but requires continuous biocuration by domain experts to keep the underlying rule set up-to-date. Rules-based WGS-AST is expected to miss novel resistance mechanisms not yet described in the literature. In contrast, machine-learning-based approaches avoid manual collection of domain knowledge; instead, an ML algorithm identifies mechanisms of AMR directly from a large dataset of sequenced isolates with known resistance phenotypes. The main prerequisite is a sizeable dataset to enable the ML algorithm to train accurate models. A major advantage of ML-based WGS-AST is that little expert biocuration is required (at the cost of clinical interpretability); the algorithms automatically pick new emerging modes of AMR resistance from new WGS-AST data.

ML-based WGS-AST typically uses nucleotide k-mer representations of either input genome assemblies or raw sequencing reads [14,15,16,17,18]. K-mer sets have been successfully used for various bioinformatics analyses, ranging from species identification [19] to genome assembly [20], as they offer advantages in computing efficiency and speed. To train a predictive AST model, an ML algorithm searches for correlations between a biological phenotype of interest and the presence of individual DNA k-mers. The dimensionality of DNA k-mer based feature spaces is typically orders of magnitude larger than the number of training samples. Training predictive models on such datasets requires careful regularization of the training process to avoid training set overfitting and poor model generalization. ML algorithms might not be able to detect biologically relevant signals in high dimensional training data or might incorrectly pick spurious correlations. The engineering of biologically relevant features pertaining to raw genomic sequences might thus be advantageous. For example, gene annotations translated into amino acid (AA) k-mers might more efficiently capture missense mutations and ignore silent mutations [21].

A key challenge for WGS-AST is that the presence of different point mutations can contribute to resistance. Different loss-of-function mutations in a single gene can decrease susceptibility, ultimately leading to resistance. In classical DNA or AA k-mer representations, resistance-contributing mutations result in the presence of different k-mers represented as different features, even though all cause a loss of function of the same causative gene. In a small training dataset, many such relevant k-mers are either missing or present in a few samples only and thus difficult to detect. Combining different mutations of a single gene or protein into a single aggregated score for each protein of interest addresses this challenge. Any disrupting mutation of a given gene would ideally generate a similar disruption score, despite being located on different positions. There are several bioinformatics tools that predict the structural effects of protein mutations and their usage in the context of AMR has been recently reviewed by Tunstall et al. [22]. Many of these tools have been used to evaluate mutations on limited sets of proteins, but to the best of our knowledge, this is the first systematic proteome-wide application of mutation scoring for WGS-AST. An efficient and robust mutation-scoring tool is needed to score millions of mutations across thousands of reference proteins within a reasonable run time and costs. The tool PROVEAN [23,24], evaluated in this study, generates predictions for approximately 10 mutations per second per the CPU core.

The performance of ML models can be improved in several ways. While the addition of training data is the most straightforward strategy, it is not always feasible. Previously, we explored the utility of different ML algorithms and their possible combinations [17]. In this study, we investigated improvements of WGS-AST ML models via the feature-space engineering approach. We evaluated whether extending a naïve DNA k-mer feature space with additional biologically relevant features, in particular automated mutation scoring, improves the predictive power of WGS-AST ML models. It is of note that a complementary strategy to improve the feature space of WGS-AST ML models is to focus on biologically relevant signals for AMR resistance phenotype determination, for example, by restricting the feature space to genes known to be associated with a given phenotype prior to model training. Such biologically informed WGS-AST models, including rule-based systems, were not considered in this study in order to focus on an exhaustive and purely statistical ML approach.

## 2. Results

### 2.1. Overview of the Data

Whole genome sequencing data together with resistant/susceptible phenotypes for nine clinically relevant pathogens and 30 antimicrobial compounds were used in this study. Altogether 110 datasets were prepared according to the protocol described in Materials and Methods section. Between 280 and 3681 isolates were used for training with a median training set size of 1368 isolates. Supplementary Table S1 contains a detailed description of each dataset.

### 2.2. WGS-AST Predictive Models on DNA K-Mers

Extreme gradient boosting (XGB) [25] models were trained on the 110 genomic k-mer datasets. The models achieved an average categorical agreement (accuracy) of 85.8%, an average major error (ME) of 11.0%, and an average very major error (VME) of 21.8%. Individual results of the DNA k-mer XGB models on the corresponding test sets are provided in Supplementary Table S2.

### 2.3. Feature Engineering

To improve WGS-AST models, we explored the utility of biologically relevant information, extending the original DNA k-mer feature space with AA k-mers, protein counts, multilocus sequence types (MLST), the presence of AMR markers, and DNA assembly quality control (QC) metrics. After the addition of the new feature types, the original DNA k-mers still made up 85% of the generated features.

On the set of 110 datasets studied, the models trained on the extended feature set achieved balanced accuracy (bACC) improved by 1.5% on average; 62 models improved, 10 models performed exactly the same, and 38 models worsened. The largest improvements in bACC were seen for models of *C. freundii* and *P. aeruginosa* with an average of 6.4% and 5.2%, respectively.

Encouraged by these improvements, the datasets were further extended with features scoring the functional alteration of protein mutations as provided by PROVEAN. Averaged over the 110 evaluated datasets, the proportion of feature types that passed the feature filtering step and were used as input for model training were split into DNA k-mers (82%), AA k-mers (12%), mutation scores (5%), protein counts (0.9%), AMR markers (0.012%), MLST types (0.0043%), and assembly QC metrics (0.0014%). Even though the mutation scores made up only about 5% of the feature space, their creation took about twice as long as the creation of all other features combined.

### 2.4. Models Trained with PROVEAN Features

**Figure 1:**
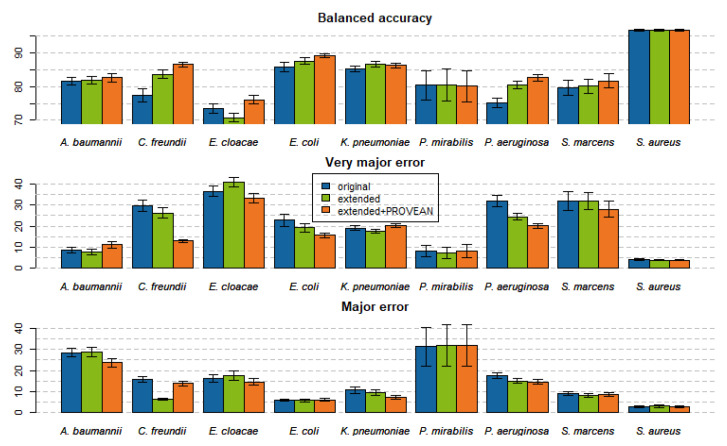
Balanced accuracy, very major error (1-sensitivity), and major error (1-specificity) of trained models as averaged across the compounds of the given pathogens. Error bars are the standard error of means.

**Figure 2:**
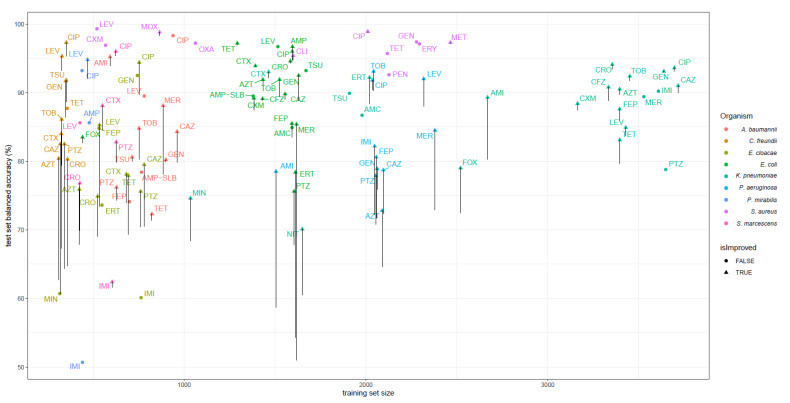
Balanced accuracy of 110 models evaluated on the 20% test set splits. The training set size is shown on the *x*-axis. For each dataset, the balanced accuracy of the best performing model of the three models considered, trained on different feature spaces (DNA k-mers, extended dataset, extended + PROVEAN features), is shown. Vertical lines indicate the increase in balanced accuracy on individual datasets from the original feature space. Section 4.2 lists all compound names and their abbreviations as shown here. A small scatter is added on the x-axis to avoid overlapping vertical lines.

Averaged over the 110 models trained, the first feature space extension improved the bACC by 1.5%. Adding PROVEAN features on top improved the bACC by an additional 1.6% on average (see Figure 1 and Supplementary Table S2). Twelve AMR models improved their bACC by more than 10% (Figure 2). Improvements in VME and ME are shown in Supplementary Figures S1 and S2.

Training models on fully extended feature spaces most improved the models for *C. freundii*, *P. aeruginosa*, and *E. coli*; their bACC improved on average by 9.3%, 7.5%, and 3.5%, respectively. The total number of errors on the test sets of these three pathogens was reduced from 2170 to 1704. Twelve of these models improved significantly, with a *p*-value < 0.05 as tested by McNemar’s test. The difference on the aggregated confusion matrix of all models of these three pathogens was highly significant (*p*-value = 5.6 × 10^−8^, McNemar’s test). The improvements on *C. freundii*, *P. aeruginosa*, and *E. coli* models were mostly driven by sensitivity improvements, reducing VME by 16.8%, 11.9%, and 7.2%, respectively. The models of the other six pathogens seem to be less affected by the addition of the new features; their VME and ME decreased on average by only 0.2% and 2.3%, respectively. The details of particular models performance are provided in Supplementary Table S2. Out of all 110 models, 19 improved significantly and 5 downgraded significantly, at significance 0.05 according to McNemar’s test. The five downgrading models achieved a bACC lowered by 1.8% on average, while the 19 significantly improved models had on average a bACC higher by 10.8%. The two models that dropped most in performance after the addition of PROVEAN features were models of cefepime and gentamicin resistance on *E. cloacae.* For both of these models, training on the fully extended feature spaces slightly decreased the number of false positives but at the same time the number of false negatives increased comparably. As the test sets of these two models contained only 32 and 36 resistant samples, the drop of the models’ sensitivities was higher than the small improvements in specificities, thus bACC dropped by about 8%.

### 2.5. Feature Importance

**Figure 3:**
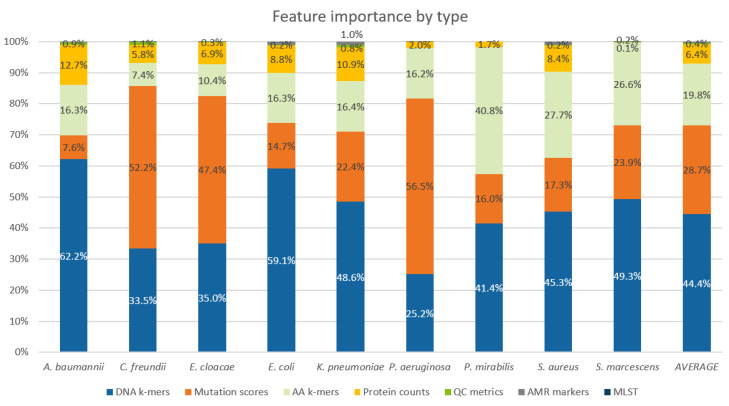
Relative importance of different feature types for individual pathogens as measured by SHAP contributions of features to model output. Values were summed up across all tested compounds.

Even though PROVEAN features made up only about 5% of all features, for the models of *P. aeruginosa*, *C. freundii*, and *E. cloacae,* the mutation scores had about 50% contribution to the outcome of the new models. QC assembly metrics, MLST types, and AMR markers contributed little to the predictions (Figure 3).

**Figure 4:**
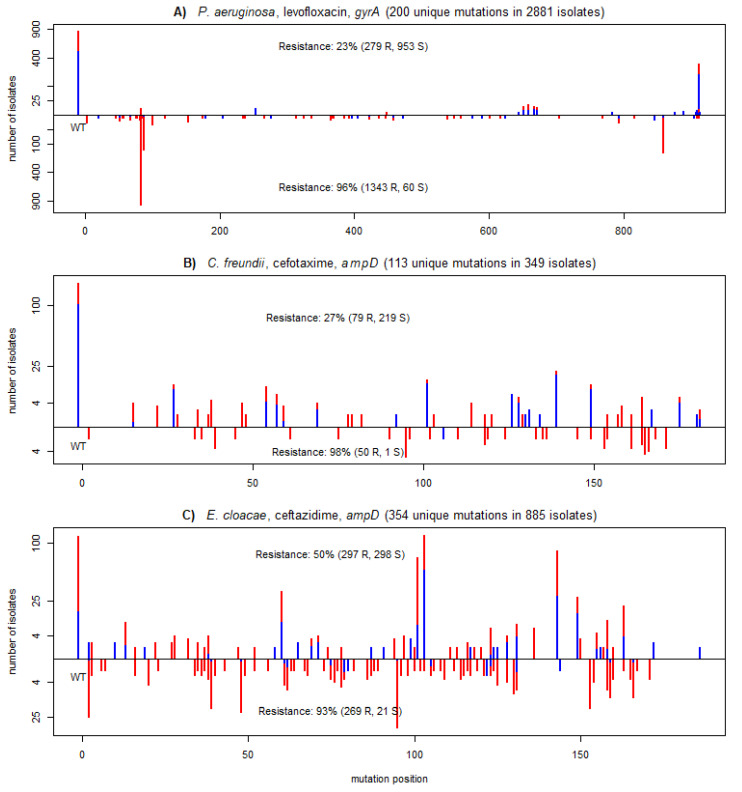
Mutational profiles across the three most important genes of the models for the following combinations: (**A**) *P. aeruginosa*, ciprofloxacin; (**B**) *C. freundii*, cefotaxime; and (**C**) *E. cloacae*, ceftazidime. Each plot shows the positional distribution of mutations of the most important gene for the model prediction. The bars show the number of assemblies having a given mutation at a given position, but only considering the most damaging mutation per assembly. Damaging mutations (PROVEAN scores lower than a gene specific threshold used by XGB models) are shown on the negative y-axis section; neutral mutations (score larger than or equal to the threshold) are shown in the positive y-axis section. The y-axis is shown in a square root scale. Red and blue colors correspond to assemblies resistant and susceptible to the given compound. The number of isolates identified as damaging or neutral along with the percentage of resistant isolates are shown for each group. Isolates with wild-type sequences are positioned at position -10 on the x-axis and labelled WT.

Figure 4 demonstrates the predictive power of protein-wise aggregated PROVEAN scores on three XGB models that achieved a high improvement of balanced accuracy. The figure shows the distribution of mutations of *gyrA* and *ampD,* known markers for antimicrobial resistance in these pathogens [26,27]. PROVEAN scores on these genes were picked by XGB as the most important features of the respective models. Both genes had more than a hundred unique mutations, many of which occurred only several times in the provided datasets. As shown on the figure, the isolates with low PROVEAN scores for the corresponding gene were mostly resistant with strong positive predictive values of 96%, 98%, and 93%, respectively. These splits not only had a strong PPV but also were biologically meaningful and made the underlying models more interpretable.

The model for meropenem in *E. coli* improved most with regard to bACC after the addition of PROVEAN features. The original model detected only a single resistant isolate in the test set. Inclusion of the first feature space extension lowered VME a little, but the inclusion of PROVEAN scores lowered it further to 28.8%. The most important features of this model were the gene length of *ompC*, the presence of *aacA1*, and the length of *ompF*. Both membrane proteins OmpC and OmpF as well as aminoglycoside acetyltransferase *aacA1* are implied in antimicrobial resistance in *E. coli* [28,29,30]. In the test set, 58% of resistant isolates had OmpC shorter than 374 AA, while only 6% of susceptible isolates had such a short OmpC. The length of OmpC seems to be an informative signal for its truncation that might be harder to pick by DNA or AA k-mer features as the truncation can possibly happen on different sites.

## 3. Discussion

Eighty AMR models distributed across all nine pathogens benefitted from the feature space extensions attempted in this study. In principle, if all possible mutations are present in a training dataset in statistically large enough quantities, DNA k-mer representation is powerful enough to pick the phenotype causative mutations. However, current WGS-AST datasets are limited in size; thus, engineered features that aggregate information across all possible mutations, e.g., from PROVEAN mutation scoring, provide advantages. Our findings confirm that for many pathogens, insufficient data are available for decision tree-like algorithms to confidently determine the relevant phenotype causing mutations from DNA k-mer feature space alone. DNA k-mers, mutation scoring features, AA k-mers, and protein counts had the highest importance in the trained models. Other considered feature types, among which were also indicators of the presence of AMR markers, had under 1% contribution in all pathogens. This suggests that k-mer based features have enough power to accurately represent presence of AMR markers and implicitly annotated presence of markers does not contribute strongly to the trained ML models, at least not without expert insight about marker-phenotype correlations as is done in rules-based models.

We hypothesize that mutation scoring can effectively combine signals from different mutations of a given protein to provide features that have a strong predictive value even if individual mutations are present in low numbers. The mutagenesis landscape of individual genes can be complex with hundreds of different genotype-altering mutations. It is a non-trivial task to determine the causality of a specific mutation if it only occurs a few times in the training data as it is likely to co-occur with several other mutations. In the most extreme case, classical k-mer-based classifiers are expected to fail on mutations that were not seen in the training set. Automated mutation scoring has the advantage that it can estimate functional effects on mutations that were not seen in the training set, contingent on the accuracy of the underlying mutation scores. Our findings suggest that this is likely the case for many genes involved in antimicrobial resistance. Any signal to be picked by an ML algorithm from the original DNA k-mer feature space can only be extracted from the labeled training WGS-AST dataset. Contrary to the k-mer counts, the extended feature sets and especially the engineered PROVEAN mutation scoring is based on data from a much larger database of non-redundant sequences used by BLAST across many different organisms. This is a practical example of the utilization of a large corpus of unlabeled data for supervised learning, a strategy successfully used in machine learning [31].

Our results demonstrate that the addition of PROVEAN scoring features or, in general, any biologically relevant metadata can be beneficial for building WGS-AST models. In particular, models may improve for pathogens that either have complex resistance phenotypes that cannot be explained by a small number of specific mutations [32], or have higher baseline mutational rates and can acquire more mutations in general, e.g., hypermutator phenotypes. The usage of the biologically meaningful feature spaces has an additional advantage of high clinical interpretability of model decisions. Further research into the utility of these engineered features is needed to fully understand their role in WGS-AST models. The current study has explored their benefit across a large set of models without any model specific optimizations.

The results presented in this study constitute a small but significant contribution towards the overall goal of improving patients’ treatment outcome in the clinic via the application of WGS-AST models. Further research into model stability, confidence, interpretability, and how to best apply predictive models in the clinic is required. A successful implementation of a WGS-AST system will likely also incorporate relevant biological knowledge of rules-based models.

## 4. Materials and Methods

### 4.1. Data Retrieval

Genome assemblies and associated resistance/susceptibility profiles for nine clinically relevant pathogens (Acinetobacter baumannii, Citrobacter freundii, Enterobacter cloacae, Escherichia coli, Klebsiella pneumoniae, Proteus mirabilis, Pseudomonas aeruginosa, Serratia marcescens, and Staphylococcus aureus) were obtained from ARESdb [13], which contains WGS and AST data from proprietary and public sources [3,8,16,33,34,35,36,37]. Compound names imported from public data sources were corrected via regular expression base matching as defined in Supplementary Table S3. Minimum inhibitory concentration (MIC) values were translated into susceptible/intermediate/resistant (S/I/R) interpretative categories via clinical breakpoints according to CLSI 29 standards [38]. Intermediate phenotypes were treated as resistant. In cases where the lowest measured MIC value was equal to the intermediate MIC breakpoint, the value was interpreted as susceptible. Sequencing reads were quality trimmed and filtered using Trimmomatic v0.39 [39] and de novo assembled using SPAdes v3.13.1 [20]. Completeness of the assembled genomes was assessed using BUSCO v5.2.2 [40] and QUAST v5.0.2 [41]. Assemblies which did not meet the QC criteria of N50 ≥ 5000, L50 ≤ 500, BUSCO completeness ≥ 75%, and BUSCO duplication ≤ 7% were filtered out. Organism–compound datasets with fewer than 100 susceptible and 100 resistant isolates were excluded. Filtered datasets were partitioned into training and test sets (80%:20%) using a genome-distance-based method [17]. This dataset partitioning method is designed to reduce similarity between the training and the test dataset. Datasets that did not contain at least 10 resistant and at least 10 susceptible isolates in the test set were excluded from the study. A detailed list of publicly available samples used for training and testing of particular models is provided in Supplementary Table S4.

### 4.2. Antimicrobial Compounds

The WGS-AST data are listed in Section 4.1 for the following 30 antimicrobial compounds that passed the data extraction criteria: amoxicillin and clavulanic acid (AMC), amikacin (AMI), ampicillin (AMP), ampicillin and sulbactam (AMP-SLB), aztreonam (AZT), ceftazidime (CAZ), cefazolin (CFZ), ciprofloxacin (CIP), clindamycin (CLI), ceftriaxone (CRO), cefotaxime (CTX), cefuroxime (CXM), ertapenem (ERT), erythromycin (ERY), cefepime (FEP), cefoxitin (FOX), gentamicin (GEN), imipenem (IMI), levofloxacin (LEV), meropenem (MER), meticillin (MET), minocycline (MIN), moxifloxacin (MOX), nitrofurantoin (NIT), oxacillin (OXA), benzylpenicillin (PEN), piperacillin and tazobactam (PTZ), tetracycline (TET), tobramycin (TOB), and sulfamethoxazole and trimethoprim (TSU).

### 4.3. Machine Learning Feature Generation

The following feature types were used for XGB models: nucleotide k-mers, amino acid k-mers, protein occurrence counts, multilocus sequence types, AMR marker presence, and assembly quality control metrics. DNA k-mers of a length of 21 nucleotides were obtained by KMC 3.1.0 [42]. To obtain AA k-mers and protein counts, PROKKA v1.14.1 [43] was run on each genomic assembly file and the resulting FASTA files were converted to AA-kmers of length 16 and protein presence count matrices. MLSTs were obtained from ARESdb and encoded using one-hot encoding. The following features were used as assembly QC metrics: the number of contigs, the length of the largest contig, the assembly length, GC content, N50, N75, L50, and L75. AMR markers in ARESdb [13] were clustered with CD-HIT [44] (parametrized as -c 0.9 -n 5 -aS 0.8 -aL 0.8). Each assembly was searched for against the database of AMR markers in ARESdb and marker clusters associated with found markers were noted. The presence of clusters was encoded in a one-hot feature encoding. Presence indicators of known AMR markers were considered as additional features; however, unlike rules-based models, no prior information about any marker-phenotype relations was provided for model training. Any existing marker-phenotype correlations had to be picked automatically by the ML algorithm purely from the training data, in the same fashion as for any other features. To exclude any possibility of biases introduced by common feature selection on the full dataset, all features for prediction on the test sets were generated separately only at the prediction time.

### 4.4. Proteome-Wide Scoring of Functional Alterations

On top of the features listed in the previous section, 4 specific mutation scores based on PROVEAN [23] predictions were determined for each protein cluster in the training dataset. Training set genomes were first annotated by PROKKA and clustered by the name of the identified gene. All identified proteins of a given name were then clustered with CD-HIT [44] (with parameters -c 0.9 -aL 0.9 -aS 0.9 -n 5) to obtain a set of protein clusters. In each protein cluster the reference sequence was defined as the most frequent sequence of the given cluster. Let *p* be a protein sequence belonging to a cluster *Cj* with a reference sequence *r_j_*. For each such a protein *p*, a set of its amino acid variants against the reference sequence *r_j_* in HGVS nomenclature [45] was determined using a tool called Mutanalyzer [46]. Let us name that set of variants *HGVS(p, r_j_)*. The union of all variants of the reference sequence *r_j_*, $\cup_{p}\mathrm{HGVS}(p,r_{j})$, was then scored using PROVEAN. We defined a score *M(p)* of the protein *p* that belonged to the cluster with the reference *r_j_* as

(1) $$M\left(p\right)=\min_{x}\left\{\;\mathrm{PROVEAN}\left(x,r_{j}\right)\;|\;x\in \mathrm{HGSV}\left(p,r_{j}\right)\;\right\}.$$

For any assembly *A_i_* and any sequence cluster *C_j_* the following four scores are then defined:

(2) $$S_{ij}^{\min}{=\min}_{p}\left\{\;M\left(p\right)\;|\;p\in A_{i}\;\wedge \;p\in C_{j}\;\right\}$$

(3) $$S_{ij}^{\max}{=\max}_{p}\left\{\;M\left(p\right)\;|\;p\in A_{i}\;\wedge \;p\in C_{j\;}\right\}$$

(4) $$L_{ij}{=\max}_{p}\left\{\;\left|p\right|\;|\;p\in A_{i}\;\wedge \;p\in C_{j}\;\right\}$$

(5) $$N_{ij}=\left|\left\{\;p\;|\;p\in A_{i}\;\wedge \;p\in C_{j}\;\right\}\right|$$

If the set {$p|\;p\in A_{i}\;\wedge \;p\in C_{j}\}\;$ was an empty set then we defined each of the above terms as zero. The above four terms (2)–(5) for each cluster can be respectively interpreted as a mutation score of the most altered protein, a mutation score of the most native-like protein, the longest protein in the cluster, and the number of proteins in the cluster. If there were *N* clusters found by CD-HIT in the training set then the above procedure defined *4N* numeric features for each assembly. The most expensive part of the mutation scores calculations was running PROVEAN itself. The computing costs can be reduced significantly if the union of all mutations of a given cluster are calculated first and then evaluated together in a single PROVEAN call. PROVEAN scores mutations by searching the reference sequence against the non-redundant BLAST database [47]. PROVEAN version 1.1.5 together with BLAST version 2.2.31 and the corresponding BLAST non-redundant database were used in this study. These BLAST searches were the most expensive part of the training data generation, but importantly the BLAST results could be cached at training time and reused at prediction time. This allowed the generation of predictions within seconds, especially since only a small subset of CD-HIT clusters needed to be evaluated at prediction time.

### 4.5. Feature Filtering

The feature matrix was prepared considering all training assemblies of a given pathogen. Zero-variance training features were removed and features with identical values across all training samples were de-duplicated, keeping one representative. Subsequently, for each organism and antimicrobial compound, a subset of the organism’s full count matrix for which S/R class information of the given compound was available was extracted. The feature space was then condensed by univariate feature selection before training. Features were tested for independence from the S/R category using the χ^2^ test as implemented in scikit-learn. The resulting *p*-values were corrected for false discovery by the Benjamini–Hochberg procedure [48] and filtered by a q-value threshold of 0.05. For *P. aeruginosa* datasets, relatively few features were correlated with phenotype at the default q-value threshold, thus the q-value threshold for *P. aeruginosa* datasets was increased to 0.2. Of the features passing the filtering steps, at most half a million features with the highest log-odds ratio were retained.

### 4.6. Model Training

Extreme gradient boosting was used for training predictive models of antimicrobial resistance from WGS data for a set of nine clinically relevant pathogens. XGB models were trained using XGBoost 1.2.0 via the provided Python 3 bindings. Only the training datasets were used for training. All models were trained with the following XGB hyperparameters: min_child_weight: 0, max_depth: 14, gamma: 0.0001, subsample: 0.721, colsample_bytree: 0.4947, colsample_bylevel: 0.5366, max_delta_step: 2, lambda: 2.394, learning_rate: 0.0485, n_estimators: 360, objective: binary:logistic, eval_metric: logloss. To counter label imbalance in the training dataset, the training isolates were weighted with weights calculated such that the total weight of susceptible and resistant isolates was equal, capped to the range 0.1 and 10 for strongly imbalanced datasets. To ensure model stability, the number of estimators in each model was controlled via the early stopping strategy by monitoring performance in an internal tenfold CV of the training set, with the early_stopping_rounds parameter set to 15. The final model was then trained on the complete training set with the number of estimators determined from the early stopped CV run. The above hyperparameters were optimized by several rounds of automated hyperparameter search using a genetic search algorithm combined with manual optimization on a selected subset of several *K. pneumoniae* and *P. aeruginosa* models. During the hyperparameter search, the XGB model performance was found to be reasonably robust to hyperparameter changes and models trained with hyperparameters optimized for individual models of *P. aeruginosa* and *K. pneumoniae* were found to have a similar performance to the models trained with the default set of hyperparameters (data not shown). The performance of trained models was evaluated on the test datasets; the internal tenfold CV was not used for model evaluation in any way. Feature Importance Scoring: The importance of individual model features was evaluated by SHAP values [49]. For each test set assembly, SHAP values for the 20 most important features were recorded. Relative importance of different feature types in individual pathogens was determined by aggregating the SHAP values over all test assemblies of all the pathogens’ models (Figure 3).

## Data Availability

Not applicable.

## Supplementary Materials

The following are available online at https://www.mdpi.com/article/10.3390/ijms222313049/s1.
