# Supplementary material for: Genome-Wide Mutation Scoring for Machine-Learning-Based Antimicrobial Resistance Prediction
Source: Int J Mol Sci. 2021 Dec 2;22(23):13049. doi: 10.3390/ijms222313049 (PMC8657983; doi:10.3390/ijms222313049)

Supplementary Figure S1.

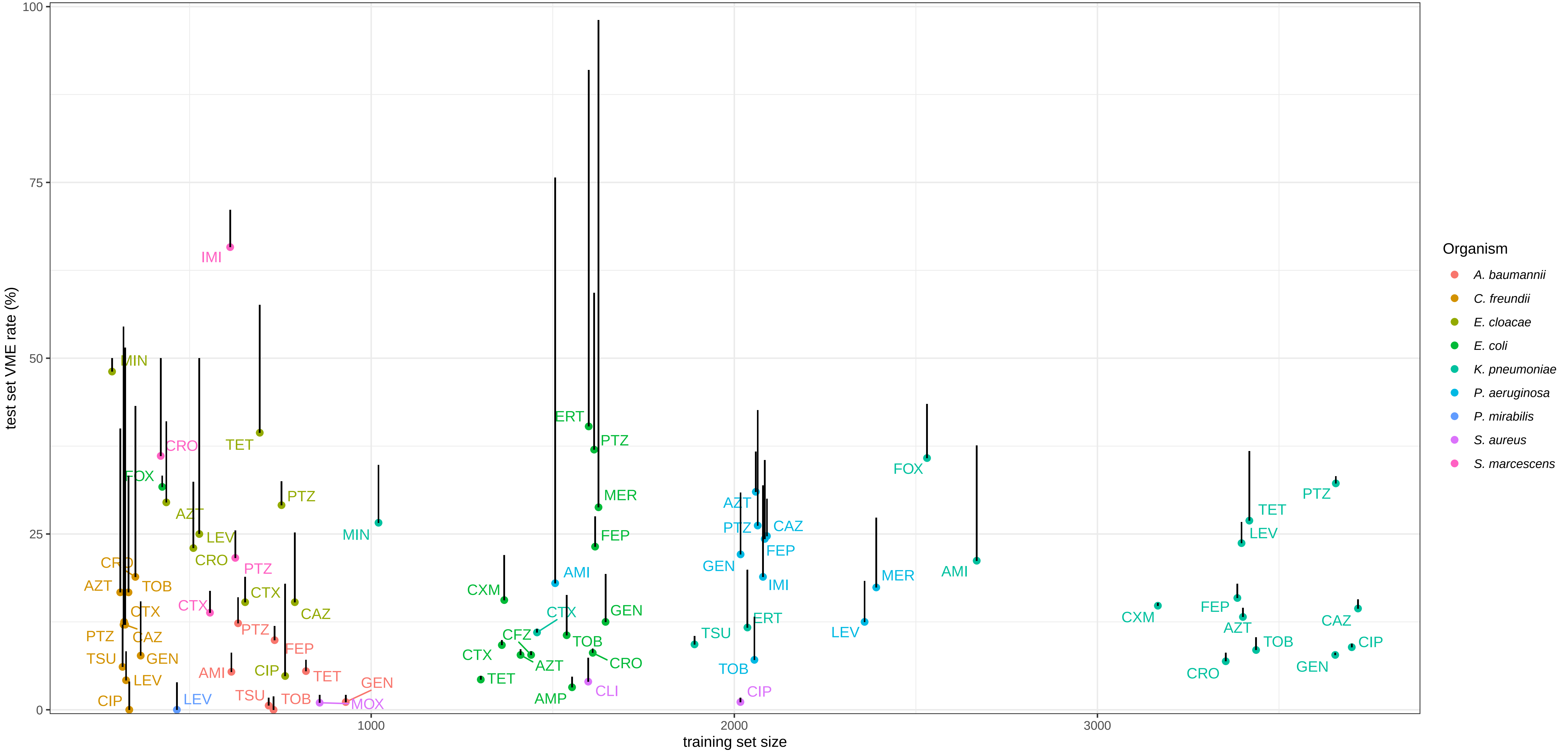

Supplementary Figure S2.

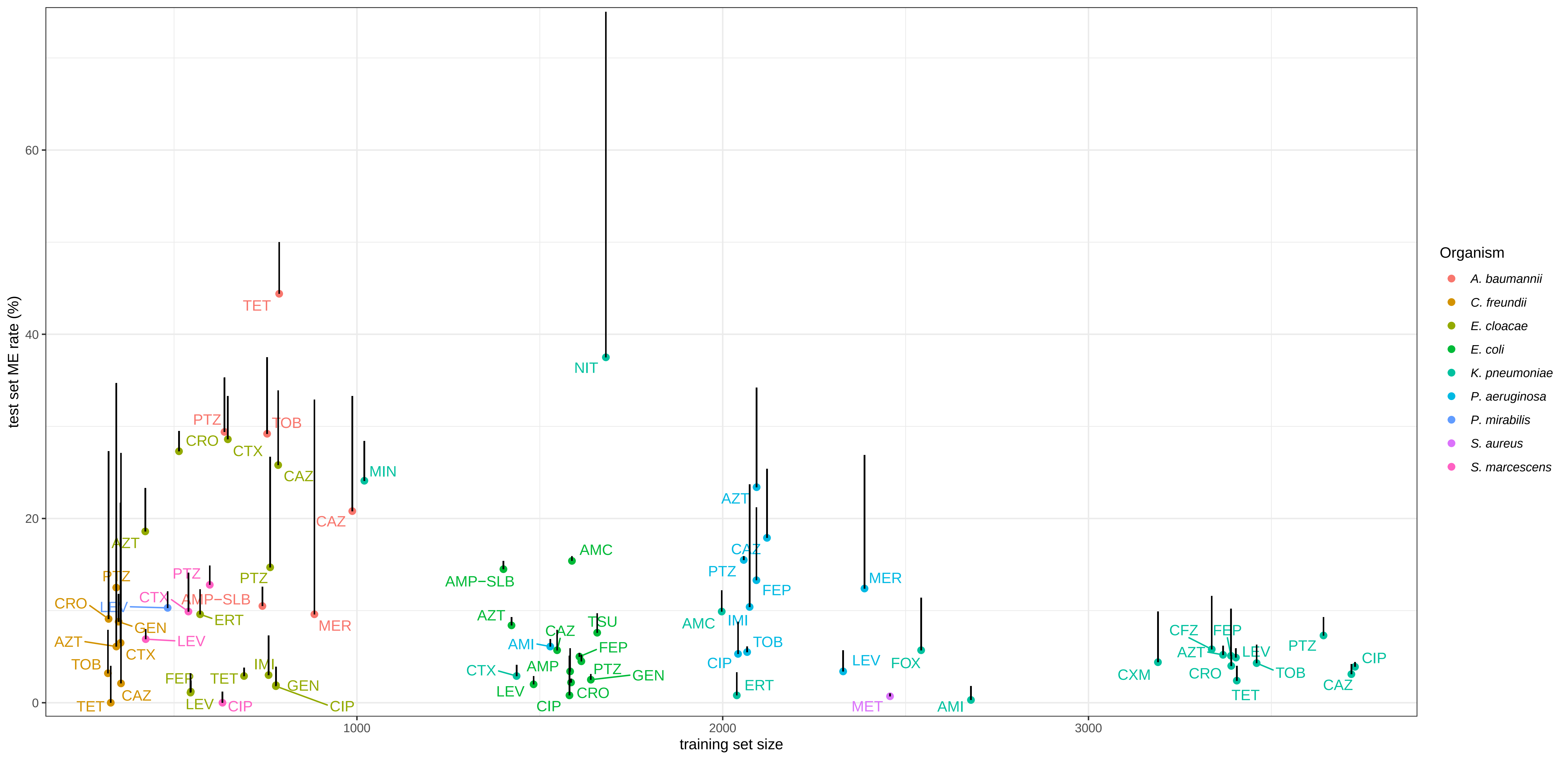

Supplement: Supplementary file 1 [file ijms-22-13049-s001.zip › ijms-1443669-supplementary/Supplementary_Files/Supplementary_Figures.pdf]
